# Dynamical differential expression (DyDE) reveals the period control mechanisms of the Arabidopsis circadian oscillator

**DOI:** 10.1371/journal.pcbi.1006674

**Published:** 2019-01-31

**Authors:** Laurent Mombaerts, Alberto Carignano, Fiona C. Robertson, Timothy J. Hearn, Jin Junyang, David Hayden, Zoe Rutterford, Carlos T. Hotta, Katherine E. Hubbard, Marti Ruiz C. Maria, Ye Yuan, Matthew A. Hannah, Jorge Goncalves, Alex A. R. Webb

**Affiliations:** 1 Luxembourg Centre for Systems Biomedicine, University of Luxembourg, Belvaux, Luxembourg; 2 Department of Plant Sciences, University of Cambridge, Cambridge, United Kingdom; 3 Department of Engineering, University of Cambridge, Cambridge, United Kingdom; 4 BASF Agricultural Solutions Belgium NV, Gent, Belgium; University of California Irvine, UNITED STATES

## Abstract

The circadian oscillator, an internal time-keeping device found in most organisms, enables timely regulation of daily biological activities by maintaining synchrony with the external environment. The mechanistic basis underlying the adjustment of circadian rhythms to changing external conditions, however, has yet to be clearly elucidated. We explored the mechanism of action of nicotinamide in *Arabidopsis thaliana*, a metabolite that lengthens the period of circadian rhythms, to understand the regulation of circadian period. To identify the key mechanisms involved in the circadian response to nicotinamide, we developed a systematic and practical modeling framework based on the identification and comparison of gene regulatory dynamics. Our mathematical predictions, confirmed by experimentation, identified key transcriptional regulatory mechanisms of circadian period and uncovered the role of blue light in the response of the circadian oscillator to nicotinamide. We suggest that our methodology could be adapted to predict mechanisms of drug action in complex biological systems.

## Author summary

Circadian oscillators are internal timing devices that produce rhythms with a period of about 24 hours to regulate a wide range of biological processes for nearly all organisms. Circadian oscillators adjust phase and period in response to external cues such as light and temperatures and internal cues such as metabolites to maintain synchrony with the daily timing of light and dark cycles resulting from Earth’s rotation. We investigated the dynamical response of the *Arabidopsis thaliana* circadian network to nicotinamide, a metabolite that lengthens the period of circadian rhythms, to uncover key transcriptional mechanisms required for the adjustment of daily biological activity. The identification of the mechanisms of drug response is challenging, as a complex cascading effect causes large section of the transcriptome to be differentially expressed, despite not being directly affected by the drug. To identify the source of the change in circadian period, we introduce a modelling strategy based on the identification and comparison of gene regulatory dynamics before and after the perturbation occurred. The Dynamical Differential Expression (DyDE) methodology uses a reverse engineering approach that favours both the identification of unknown Gene Regulatory Network (GRN) topology and the interpretation of the possible dynamical changes, without the need to cover extensive experiments or to make prior assumptions of network dynamics. Subsequently, we show that our methodology can reliably identify the source of a perturbation in complex regulatory systems such as the circadian network. The proposed mathematical framework is scalable and flexible, so that it can be applied to large datasets with scarce sampling.

## Introduction

The synchronization of physiological rhythms with the external environment is important for nearly all organisms. Circadian oscillators are internal timing devices that produce rhythms with a period of about 24 hours to regulate a wide range of biological processes. Circadian rhythms maintain synchrony with the daily timing of light and dark cycles resulting from Earth’s rotation by constantly integrating environmental signals. This process of synchronization is called entrainment. Studying the mechanisms that dynamically adjust circadian period and phase, therefore, is critical to understand the control of daily biological activities.

In *Arabidopsis thaliana*, the circadian oscillator consists of a complex circuit of highly connected transcriptional regulators. Together, they coordinate global transcript accumulation and diverse biological processes, such as photosynthesis, hormone signaling, hypocotyl elongation and plant-pathogen interactions [1,2,3,4,5]. The light perception of the circadian oscillator is conferred by a suite of photoreceptors. The photoreceptors are split into two classes: phytochromes (principally *PHYA* and *PHYB*), that primarily sense the red portion of the spectrum [6] and cryptochromes (*CRY1* and *CRY2*) that are sensitive to blue light [7,8,9].

Recent studies have demonstrated a role for metabolism in regulating and entraining the circadian oscillator of *Arabidopsis thaliana*. The primary metabolite sucrose accelerates the circadian oscillator (i.e., reduces its period) through regulation of the morning expressed gene *PSEUDO RESPONSE REGULATOR (PRR) 7* [10], while *GIGANTEA* (*GI*) has been identified as a necessary sucrose-signaling mediator in the dark [11]. Another metabolite, nicotinamide (NAM), a breakdown product of nicotinamide adenine dinucleotide (NAD), causes long period of the circadian oscillator in all organisms tested [12,13]. The mode of action of NAM is uncertain: various mechanisms having been proposed, including inhibition of the production of the Ca^2+^-agonist cyclic adenosine diphosphate ribose (cADPR), inhibition of polyADP ribose polymerases and histone modifications [12,13,14]. The goal of this study was to use NAM as a tool to identify the processes responsible for a change in circadian period, which might be required for circadian entrainment and homeostatic adjustment [15,16,17].

**Fig 1:**
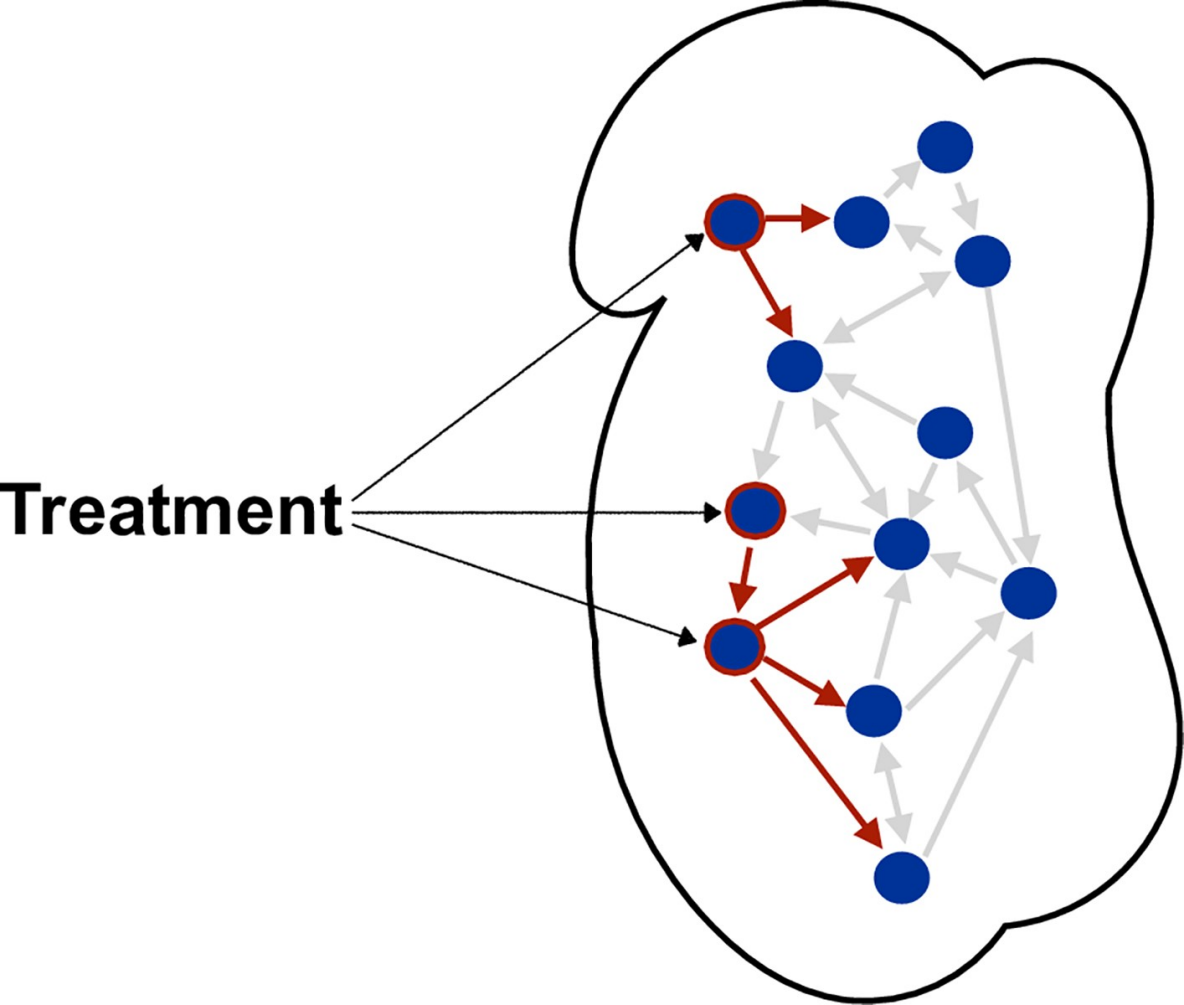
Treatments effects in transcriptional networks. Treatment effects can be perceived as perturbations in molecular networks [18,19]. In transcriptional networks, such perturbations usually only affect a very small number of regulatory links directly. For example, only the red links have been directly affected by the treatment. All other links are unchanged, although all nodes (concentrations) in the Figure have been (indirectly) affected due to cascading and feedback effects. Hence, Differential Expression (DE) might not distinguish between direct and indirect effects of a treatment. Dynamical Differential Expression (DyDE), therefore, investigates *how* and *why* changes occur, instead of simply measuring *what* and *how much* is produced by those changes.

The discovery of drug modes of action, however, is still a costly and inefficient process, which often requires considerable prior knowledge of a biological system and/or a vast amount of data in several experimental condition (e.g. mutations). A major difficulty is the complex ripple effect of treatments affecting transcriptional networks. Large sections of the transcriptome can be differentially expressed, despite not being directly affected by the treatment (off-targets) (Fig 1). Due to the large number of feedback loops involved in a complex and relatively small Gene Regulatory Network (GRN), such as the circadian clock, this effect is particularly significant as a perturbation anywhere in the network typically strongly affects all molecular concentrations. Additionally, as the perturbations induced by NAM in the circadian clock are intrinsically related to changes in circadian period, a large part of the transcripts will typically be differentially expressed. Thus, Differential Expression (DE) analysis, the traditional approach used to identify the mechanisms that alter biological behavior in response to drugs, environmental signals or genetic lesions [20], will usually fail to identify the small number of genes central to the biological perturbation. The main reason is that DE only performs statistical analysis of changes in gene expression levels [21,22]. As an alternative to the DE analysis, we devised a modeling framework that identifies and characterizes differentiated regulatory dynamics between genes to capture key mechanisms involved in NAM-induced perturbations in the circadian system of Arabidopsis. The rationale behind this approach is that not only genes, but also their interactions, are affected by a drug. This reasoning is further supported by [18,23,24], which highlight the fact that drugs and diseases mechanisms should be regarded as network instead of gene-centric perturbations. We designed our modelling strategy so that it could be applied to scarce data without the need to cover extensive experiments or to make prior assumptions of network dynamics. In particular, we consider only gene expression data with and without NAM.

On one hand, complex nonlinear models have the potential to capture the dynamical relationships between genes with great precision. A successful application of Michaelis-Menten dynamics to reverse engineer network topology from multiple experiments, circadian time-series data is presented and compared to state-of-the-art strategies in [25]. Alternatively, a community-driven comparison of (non)linear approaches (e.g. mutual information-based, Bayesian networks, random forests, neural-networks, etc.) for the inference of (non-circadian) gene regulatory networks has been achieved in [26,27]. On the other hand, high model complexity can lead to overfitting (fitting the noise instead of the dynamics) without sufficient data or detailed knowledge such as network topology, types of nonlinear interactions, or potentially some of the model parameters (e.g. Hill coefficients). As for non-model based methods, such as [28,29,30,31], it is not clear how they can be used to compare subtle changes in dynamics caused by perturbations, and pinpoint the source of those perturbation. We developed, therefore, a systematic and scalable dynamical modeling framework named Dynamical Differential Expression (DyDE). DyDE uses a black box-type modeling approach to reverse-engineer simple yet consistent and comparable gene regulatory dynamics from time-series data. In addition, it does not use any prior information and, hence, it is unbiased towards prior knowledge of network topology and dynamics. By comparing changes in both topology and subtle dynamic modifications of regulatory mechanisms, we were able to considerably narrow down potential targets of NAM in the circadian clock.

The findings predicted by DyDE are experimentally tested and demonstrate the role of the circadian gene *PRR7* as a key regulator of dynamics adjustment of the circadian clock. In addition, *TIMING OF CAB EXPRESSION 1* (*TOC1*) and the interplay between *PRR7* and *PSEUDO RESPONSE REGULATOR 9* (*PRR9*) are identified as the main mediators of the circadian system response to NAM.

The modeling insights also identified alterations in *CRY2* dynamics resulting from the NAM treatment. Therefore, we also investigated the role of blue light in the circadian period change of NAM-treated plants. In particular, we found that blue light regulates circadian oscillations of [Ca^2+^]_cyt_ through a NAM-sensitive pathway. These new perspectives contribute to the understanding of the mechanistic details underlying the regulation of period of circadian oscillators.

Overall, the results suggest that DyDE is a useful tool to generate reliable hypothesis from time-series data for the identification of drug targets in complex biological systems.

## Methods

To investigate how NAM might regulate the period of the circadian oscillator we first used statistical tools to identify those transcripts that have circadian rhythms in abundance in both untreated and NAM-treated plants. Then, we introduce the Dynamical Differential Expression (DyDE) approach to characterize altered dynamics within the circadian regulatory network of NAM-treated plants.

The hypothesis generated by DyDE were experimentally tested using genetic mutant and physiological experiments in different light conditions. Finally, we extended DyDE to the whole rhythmic transcriptome to further investigate clock period regulation.

### Statistical characterization of circadian transcripts

**Fig 2:**
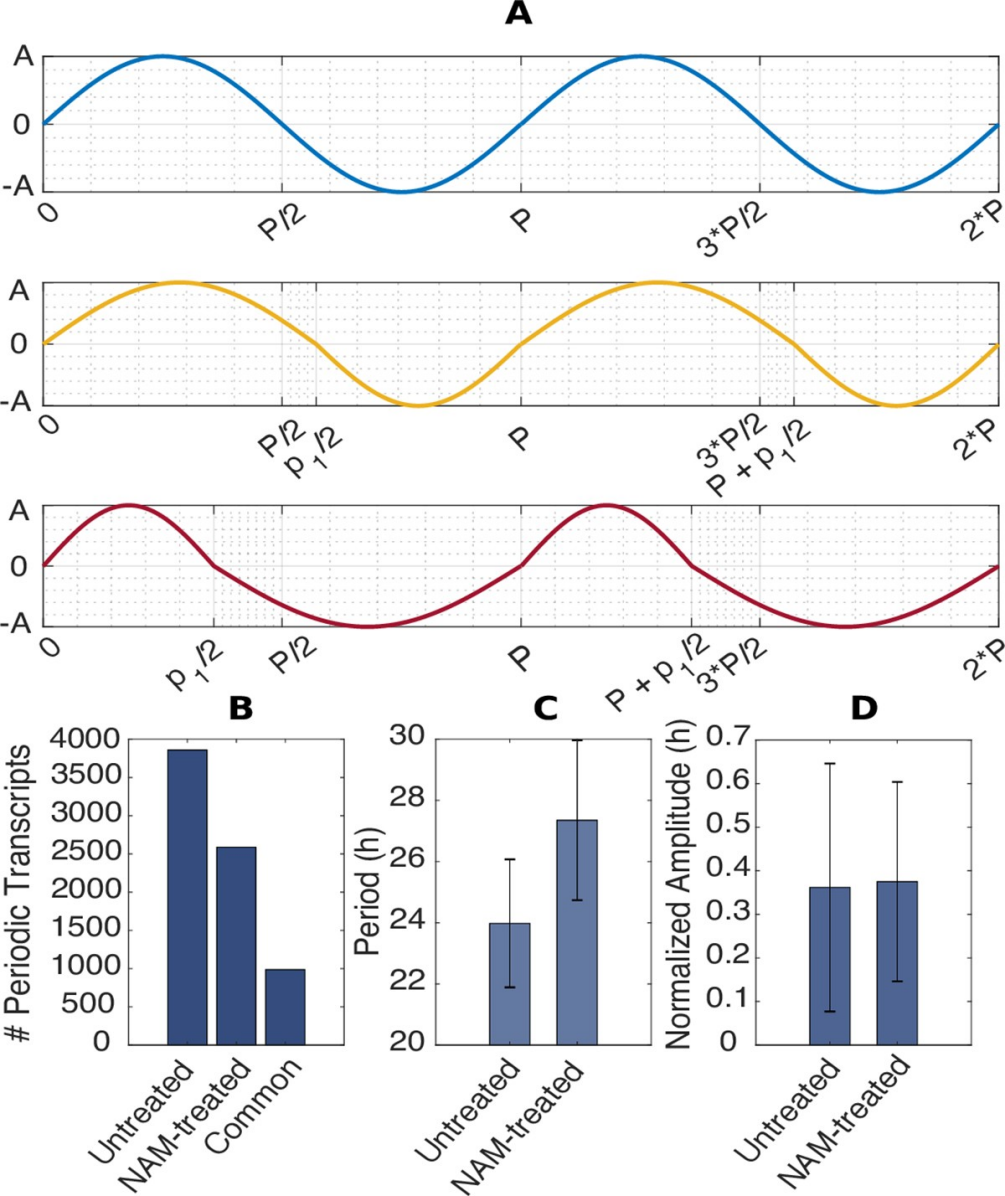
The effects of NAM on the circadian regulation of the transcriptome. **(A)** Illustration of the shape of S. The first panel shows two period of a perfect sinusoidal shape, whereas the second panel displays the segmentation of the period P into p1 and p2, where p1 is greater than p2. p1 and p2 follows the formula: P = (p1 + p2)/2. The last panel displays the case were p1 is smaller than p2. **(B)** Number of periodic transcripts that have been identified in untreated and NAM-treated plants, as well as the intersection. **(C)** Circadian period of untreated and NAM-treated transcripts plus minus standard deviation. The mean increase of period following the NAM treatment is of 3.3h. **(D)** Amplitude analysis (normalized) for the same transcripts.

To assess whether genes are regulated by the circadian oscillator, most methods take advantage that circadian regulation of transcript abundance resemble a sinusoid. To estimate circadian period of the regulation of a particular transcript, the main idea is to find the sinusoid that most closely matches its abundance over time [32,33]. However, in NAM-treated plants the changes in abundance of circadian-regulated transcripts have a considerable number of non-sinusoidal profiles (S1 Fig). To overcome this problem, we devised a learning approach based on pseudo-sinusoidal functions to properly assess the rhythmicity and the corresponding circadian period of signals from gcRMA normalized microarray data of NAM treated plants. To infer period, phase and amplitude, linear trends are eliminated by removing the best straight-line fit and pseudo-sinusoidal functions are fitted to each signal to minimize the 2-norm error. Pseudo-sinusoidal functions account for many signals that are periodic but not sinusoidal. Pseudo-sinusoidal functions are constructed by joining together two sinusoids with different periods. Hence, a complete oscillation of a pseudo-sinusoidal function consists of the first sinusoid (of period *p*_1_) in the first half-oscillation, and the second sinusoid (of period *p*_2_) in the second half-oscillation (Fig 2A). The resulting period of the pseudo-sinusoidal function is defined as $p=\frac{p_{1}+p_{2}}{2}$. This can be expressed by:

$$S=\left\{ \begin{array}{c} A*\sin(\frac{2\pi }{p_{1}}*t+\varphi_{1}),\;t\in [0,\frac{p_{1}}{2}]\\ A*\sin(\frac{2\pi }{p_{2}}*(t-\frac{p_{1}}{2}+\frac{p_{2}}{2})+\varphi_{1}),\;t\in [\frac{p_{1}}{2},\frac{p_{1}}{2}+\frac{p_{2}}{2}] \end{array}\right.$$

where *A* is a scaling factor that accounts for the amplitude of the signal and *φ*_1_ is the phase of the signal. The algorithm searches possible combinations of *p*_1_ and *p*_2_ to minimize the least square distance between pseudo-sinusoidal functions and the data. We allowed periods *p*_1_ and *p*_2_ to vary between 12 and 36 hours. A perfect sinusoid gave a high fit for the wild-type background dataset. We found that three periodic signals were highly represented in the dataset. In particular, those with *p*_1_, *p*_2_ equal to: p/2, p/2 (pure sinusoid); p/2+3.8, p/2–3.8 (p1 is greater than p2); and p/2–7.3, p/2+7.3 (p1 is smaller than p2) (Fig 2A).

We used a logistic regression framework to generate a probabilistic discriminative model that estimates the probability of a gene to be rhythmic given its time course data. In this case, the classification problem only contains two classes: rhythmic (*C*_1_) and arrhythmic (*C*_2_). For each transcript, a set of 8 features *x* = {*X*_1_,*X*_2_,…,*X*_8_} is computed and empirically believed to be crucial to distinguish between rhythmic and arrhythmic transcripts.

The features were computed from 2 signals: the first signal (A) corresponds to the average of replicates and (B) being a single replicate for which the L2-norm error with the best fitted pseudo-sinusoidal function is lower than for the other replicate. The following features were computed: ratio of power in the 18–32 hours frequency range (of (A) and (B)), L2-norm of the error to the best fit of pseudo sinusoidal function (of (A) and (B)), the variance of the power spectrum (of (A) and (B)) and the amplitude of the best fitted pseudo-sinusoidal function (of (A) and (B)).

The log of the ratio of probabilities between the two classes, also known as the log odds, is given by [34]:

$$\ln\left(\frac{p(\mathrm{rhythmic}|x)}{p(\mathrm{arrhythmic}|x)}\right)=\ln\left(\frac{p(C_{1}|x)}{p(C_{2}|x)}\right)=\ln\left(\frac{\sigma }{1-\sigma }\right)=logit(\sigma )$$

The goal of the logistic regression is to estimate *σ* for a linear combination of the *X*_*n*_ features such that:

$$logit(\sigma )=b_{0}+b_{1}X_{1}+b_{2}X_{2}+\cdots +b_{n}X_{n}$$

The weights *b*_*i*_ of the independent variables *X*_*i*_ were estimated using the *mnrfit* function in MATLAB. The algorithm is initially trained with a mix of 100 rhythmic and 100 arrhythmic transcripts randomly chosen from the dataset and visually inspected to show clear (ar)rhythmicity. Finally, the decision boundary was set so that if *p*(*C*_1_|*x*)>0.5, the gene was classified as rhythmic, and vice versa. Our approach, therefore, is inspired by the patterns observed in the dataset but not strictly constrained to pure cosine shapes. With the inclusion of the S function, we allow the search for asymmetric signals, which represent a large part of the transcriptome. A main distinction with the previously introduced algorithms, therefore, is the data-specific, learning approach devised to allow for a wider range of periodic signals. However, this offers additional advantages such as a dedicated way to handle noise between replicates, or the information in the frequency domain of the signal, which are both learned from the data. Comparison of performances with standard periodicity assessment tools is shown on S2 Fig.

### Network inference and analysis by DyDE

Like most biological systems, circadian clocks have a large number of feedback loops. Hence, a perturbation anywhere in the network typically affects all nodes (in this case, their molecular concentration and time profiles), which makes the problem of inferring the entry point of a perturbation hard using DE analysis. We proposed, instead, that key mechanisms involved in NAM-induced perturbations in the circadian system of Arabidopsis can be captured by identification and comparison of regulatory dynamics before and after the perturbation occurred. Assume that a perturbation, such as NAM, changes the regulatory dynamics between two genes (e.g. by binding to a transcription factor) while leaving intact the rest of the system. Due to feedback interconnections, all the clock genes would change their expression, which, in turn, would change the expression of all circadian genes. While thousands of genes change their expression, only one regulatory link was actually affected. Our goal is to find this link (or links, in case of multiple perturbation entry points). To achieve this, we developed DyDE that looks for changes in links, instead of nodes. DyDE uses Linear Time-Invariant (LTI) models, a type of black box model, to systematically capture the dynamics underlying the biochemical mechanisms of circadian gene regulation, without relying on *a priori* knowledge of the system or extensive database. Such models benefit from a rich theory and a well-established collection of tools that makes the analysis of its dynamical properties straightforward, as contrast to detailed mechanistic models. In addition, the estimation of the parameters of such models is reliable and computationally efficient. The description of biological mechanisms of the Arabidopsis circadian clock from time-series data by LTI models has been studied in [35]. More recently, the performances of such linear modelling approach to reverse engineer the clock topology were compared for two extensively used Arabidopsis oscillator models [36] and confirmed that the majority of oscillator links can be represented by simple linear dynamics. However, the use of LTI models to detect dynamical perturbation in the gene regulatory network resulting from chemical treatments is novel.

The first step of DyDE consists of uncovering dependencies and quantifying dynamics between genes with LTI models. Our mathematical framework estimates a collection of Single Input-Single Output (SISO) models between pairs of genes to characterize the system dynamics. The limited number of available time points restricted the modelling of SISO systems to first and second order models. Overall, second order systems did not improve significantly the fitness of models and resulted in a considerable increase of false positives (overfitting). Hence, in this analysis of the circadian system, we restrict the model order to one. Mathematically, the dynamics between two genes can be represented as:

$$\frac{dy(t)}{dt}=a\;u(t)-b\;y(t)+c$$

where *u*(*t*) and *y*(*t*) represent the time series of the regulatory gene and the regulated gene, respectively. In addition, *b y*(*t*) corresponds to the degradation rate of gene *y*, *a u*(*t*) corresponds to the influence of *u*(*t*) on the rate of *y*(*t*) and *c* is a constant offset. System identification is performed using the function ‘pem’ implemented in MATLAB to minimize the prediction error [37]. The model has a total of three parameters (*a*, *b*, and *c*), leading to efficient solutions. We chose a subspace initialization algorithm since it performed similarly as randomizing initial conditions–for the vast majority of models (99%), the final solution was identical with either method. This suggests that the chances of being trapped into a local minimum are negligible.

The estimation of parameters requires low computational time: a single system between a pair of genes is typically identified within few seconds (Intel Core i5). This modeling is independently repeated for all available pairwise genes, where each gene takes its turn as being an input and then an output to another gene. This modeling approach, therefore, generates a large amount of SISO LTI models (*n*^2^−*n* models, where *n* corresponds to the amount of genes, and self-regulation is not considered) to describe the system. Each potential link between two genes is validated if the corresponding model reproduces the dynamics involved with a sufficient degree of precision, which is characterized by a high goodness of fit, defined as:

$$fitness=100*(1-\frac{\sum_{k=1}^{N}\sqrt{{\left(y-{\hat{y}}_{k}\right)}^{2}}}{\sum_{k=1}^{N}\sqrt{{\left(y-\bar{y}\right)}^{2}}})$$

where *y* is the validation data, $\bar{y}$ is the average value of the validation data, and ${\hat{y}}_{k}$ is the estimated output. MATLAB function *compare* can be used to compute the fitness of the model. A fitness equal to 100% corresponds to a perfect identification. The choice of such metric is motivated by the dependency of noise towards the abundance of gene expression. When the distance of the true data points towards the mean is large (represented by the denominator in the above equation), the fitness conveniently penalizes less the error term, which lies in regions where the intrinsic noise involved in the gene expression is potentially the largest.

The second step consists in identifying the effect of a treatment, NAM in our case, on the biological network. While a treatment might affect the abundance of many transcripts, only a few links are affected, as depicted in red in Fig 1. Hence, checking whether links are affected before and after perturbation can potentially lead to the finding of the entry point of the treatment. For this purpose, two cases are of particular interest. First, a link between two genes is validated in the untreated system alone (i.e. it is not possible to find a combination of *a*, *b* and *c* so that the model in the treated system provides a good match with the data anymore). Second, a link is validated in both systems, but the way one gene regulates the other may change; this is a much subtler change in the dynamics of the link. The latter case requires us to compare the dynamics between both links. Here, we use a rigorous and well-established tool from engineering known as the nu-gap [38]. Originally developed to address the stability properties of closed loops systems defined in the same feedback loop, the nu-gap essentially measures the distance, from a perturbation point of view, between linear models. This property is particularly relevant in the context of circadian clock networks, which consist in regulatory networks with several feedback loops. This then facilitates us to determine the significance of the dynamical change of a link between experimental conditions. The nu-gap returns a value between 0 to 1, quantifying whether the models are similar or very different, respectively. [39] have suggested that values above ~0.2 could be used to infer the main target of a perturbation. The nu-gap is computed using the *gapmetric* function in MATLAB. It should be applied to all models that have been estimated in both networks. If the signals are concentrated around a particular range of frequencies (such as oscillating signals), the gap should be measured ‘locally’ around that range of frequencies only, since they dominated the model estimation in Step 1.

Next, we explain the key ideas behind DyDE through a small number of genes in the Arabidopsis circadian oscillator. For example, the following model considers *TOC1* as an input and *PRR9* as an output.

$$\frac{d{\left[PRR9\right]}_{t}}{dt}=a{\left[TOC1\right]}_{t}-b{\left[PRR9\right]}_{t}+c$$

where *b* represents the strength of activation or repression induced by *TOC1* on the expression rate of *PRR9*, and *a* corresponds to the degradation rate of *PRR9*. These parameters are estimated by minimizing the prediction error from the untreated time-series for both *TOC1* and *PRR9*.

**Fig 3:**
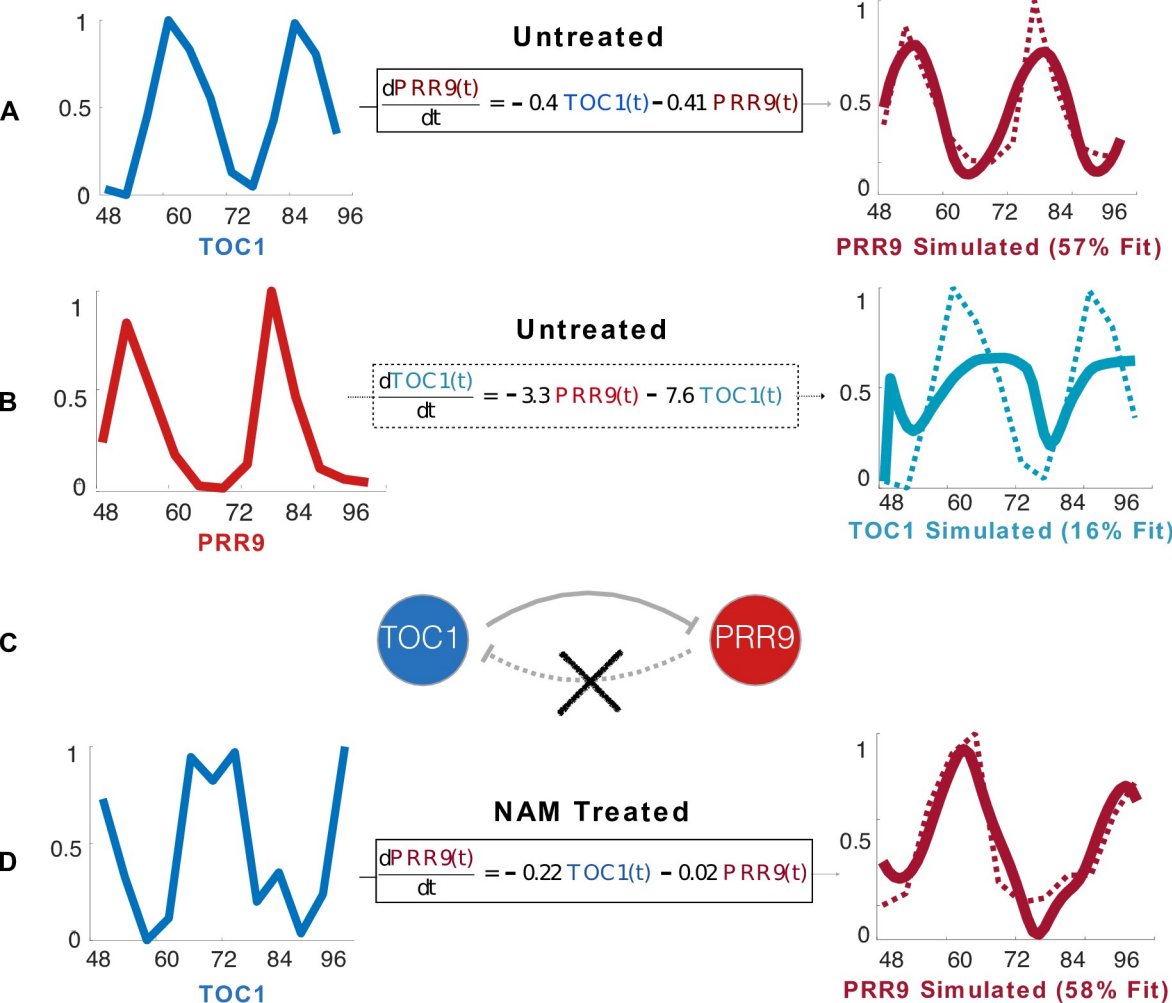
Network inference and analysis by dynamical differential expression (DyDE). **(A)** Ordinary Differential Equations (ODEs) capture the dependence of the rate of the concentration of a transcript on the concentration of another transcript. First order linear models are used to represent the dynamics between two genes. Here, a good agreement (plain line) with the data (dotted line) was found (57% goodness of fit). **(B)** The inverse regulation is considered. In this case, it is not possible to find a combination of parameters so that a first order linear model captures the dynamics involved. For this inverse regulation the model that best described the data obtained a goodness of fit of only 16%. **(C)** A threshold by which each model is (in)validated is applied on the goodness of fit of the models. As an example, a threshold of 46% would consider a link from TOC1 to PRR9 but not the other way around. The same threshold is applied to all models. **(D)** A first order linear model is evaluated in the presence of nicotinamide between the same species. The nu gap is then applied to compare models **(A)** and **(D)** to quantify whether the models are similar, or significantly affected by NAM.

In this case, we found a model in good agreement with the data (57% fitness), suggesting that indeed *TOC1* regulates *PRR9* (Fig 3A). Moreover, the model demonstrates that the rate of change of the concentration of *PRR9* is proportional to the concentration of *TOC1*. Note that the other way around (i.e., *PRR9* regulating *TOC1*) could not be established since the respective model has a low goodness of fit (16%, Fig 3B). These results are consistent with the literature [40]. Hence, we would then establish a link from *TOC1* to *PRR9*, but not the other way around (Fig 3C).

Then, a model is estimated between *TOC1* and *PRR9* from the NAM-treated time-series. From the untreated and treated time-series alone, it is unclear whether the link dynamics have changed (Fig 3D). The optimal model parameters, however, have significantly changed. A nu-gap of ~0.5 confirms that indeed the link has been affected. This result indicates that there is large perturbation in the regulatory dynamics that links *TOC1* to *PRR9*, which, therefore, should be considered as a strong candidate for being an entry point for NAM in the system. If true, knocking down either *TOC1* or *PPR9* would therefore lead to NAM no longer affecting the clock. This analysis is then repeated for all common links between untreated and treated plants.

## Results

We identified 3859 (18.4%) circadian-regulated transcripts for the untreated plants (Fig 2A). These were enriched for Gorilla terms ‘Circadian Rhythm’ and ‘Rhythmic Process’ (p = 4.07E-18; S2 Table; GEO No. GSE19271). A total of 2588 (12.3%) transcripts were identified as rhythmic in NAM-treated plants (Fig 2A), with a mean increase in period from 24.0 ± 2.1 h (-NAM) to 27.4 ± 2.6 h (+NAM) (Fig 2B) and without a noticeable change in amplitude (Fig 2C).

### DyDE applied to the Arabidopsis circadian clock genes

We considered a total of 17 known clock genes: *CCA1*, *LHY*, *PRR9*, *PRR7*, *PRR5*, *RVE8*, *GI*, *TOC1*, *ZTL*, *ELF4*, *ELF3*, *PHYA*, *PHYB*, *CRY1*, *CRY2*, *CHE and PRR3*. However, the core oscillator genes *ZTL*, *ELF3*, *PHYB*, *CRY1*, *PRR3* and *CHE* were identified as non-rhythmic in the presence of NAM, which was confirmed by visual inspection (S1 Fig). Hence, these genes are excluded from the modeling of NAM targets as they cannot be contributing to the rhythmic dynamics of the remaining oscillator components that are measured in the presence of NAM.

As a first step, we computed models for all available pairs of the clock genes for both conditions, totaling 220 SISO models (110 in untreated and 110 in NAM). We kept only those models with good agreement with the data, i.e. above a fitness threshold. On one hand, the user-defined threshold has to be set large enough to reliably capture the dynamics involved between genes, and provide the nu-gap analysis with comparable models. On the other hand, the threshold has to be set sufficiently low to consider enough gene-to-gene relationships to detect a dynamical perturbation in the network. Here, the fitness threshold was set to 46% as we noted that below this threshold, the amount of unknown regulations dramatically raised (S3 Fig; S3 Table).

In total, 70 regulatory links were retained for untreated plants and 55 links for NAM-treated plants between the 11 clock genes. The untreated models describe 70% of the known regulatory pathways among these 11 genes (S3 Table; S3 and S4 Figs [40]). 64% of which, had the expected activation or inhibition effect. These numbers are remarkable, taking into account the model simplicity, and confirms that the majority of clock links can be represented by simple linear dynamics [35,41,42].

In particular, 28 links were present in the untreated samples but not in the NAM-treated samples. These 28 links form a network from now on referred to as “regulation loss” network, which captures the links abolished by NAM. In addition, 42 links are present in both conditions which form a network, so called “common” network that is common to both treated and untreated plants (S3 Table).

**Fig 4:**
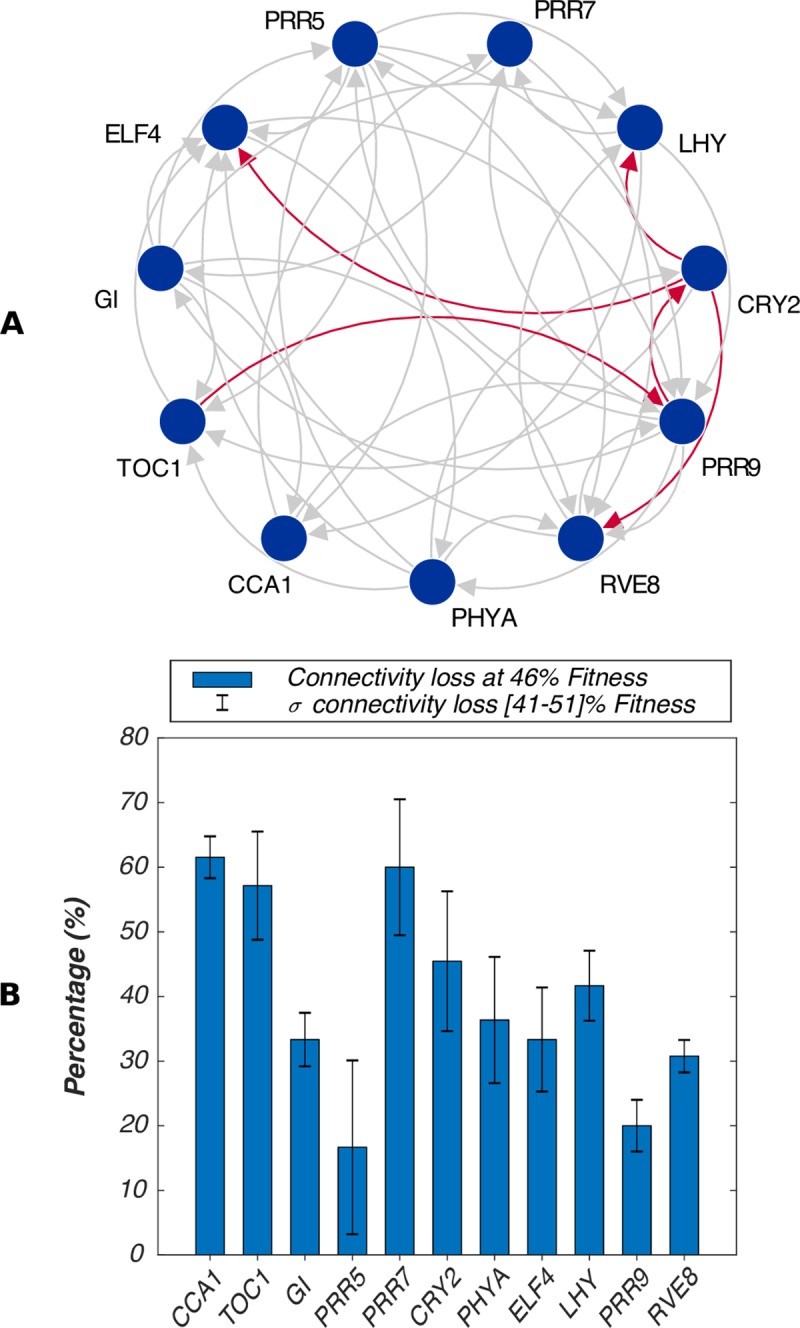
DyDE applied to the Arabidopsis circadian oscillator genes. (**A**) Common network and nu-gap analysis. The common network displays the models that have been validated in both untreated and NAM-treated plants. A directed arrow from gene *a* to gene *b* (blue circles), therefore, represents a dynamical model that captures the dependency of *b* on *a*. Red arrows represent the models associated with the top five highest nu-gap values. (**B**) Bar plot comparing the connectivity loss (%) associated to each gene. For a particular gene, the connectivity loss corresponds to the total amount of incoming and outgoing links that were validated in untreated plants but not in NAM-treated plants. Error bars represent the standard deviation of connectivity loss for ± 5% change in fitness threshold selection.

We used the nu-gap to identify those links among the common network whose dynamics were significantly affected by NAM. Fig 4A and S4 Table depict the comparison of the dynamics of each link with the nu-gap. All regulatory interactions are somehow affected by the treatment, which is expected from the interconnected circadian network. Let us then consider the highest nu-gap values, which are associated with the following links: *TOC1* to *PRR9* (0.5), those originating from *CRY2* to *ELF4* (0.47), *LHY* (0.42) and *RVE8* (0.37) and *PRR9* to *CRY2* (0.35). Interestingly, the only inferred interaction originating from *CRY2* that does not seem affected connects to *TOC1* (nu-gap of 0.06). These results suggest that a major dynamical change is induced to *CRY2* in the dynamical response of the circadian clock to NAM. In addition, the largest nu-gap value suggests that the causality within the time course data of *TOC1* and *PRR9* has changed significantly differently towards the treatment, as compared to the other parts of the circadian network.

We then used a standard network topology metric to identify the genes that are central to the drastic changes in dynamics captured by the regulation loss network. This topology metric accounts for the connectivity of a gene, i.e. the number of its incoming and outgoing links. This measure is estimated for each gene of the regulation loss network. As an example, *PRR7* has six incoming links and nine outgoing links for untreated plants. The connectivity of *PRR7* in untreated plants is then equal to 15. Among those, only six of were present in NAM-treated plants. *PRR7*, therefore, has a connectivity of nine in the regulation loss network, which correspond to a loss of 60% of its connectivity from untreated to NAM treated plants. As a result, *CCA1* (61%), *PRR7* (60%), *TOC1* (57%) exhibit the highest connectivity drop (Fig 4B; S5 Table). This result identifies the biological functions of *CCA1*, *PRR7* and *TOC1* as being highly affected by NAM in the regulation of the circadian clock.

DyDE, therefore, identifies the regulatory dynamics of *TOC1*-*CRY2*-*CCA1*-*PRR7* as being predominately affected by NAM as a result of both nu-gap and connectivity analysis. Accordingly, the strong emergence of the blue light receptor *CRY2* in the nu-gap analysis suggests that nicotinamide alters the regulation of the interactions between light signaling and the circadian oscillator. These findings are further examined through mutant analysis and single wavelength light experiments.

### *PRR7/PRR9* inter-regulation together with *TOC1* are targets of nicotinamide

**Fig 5:**
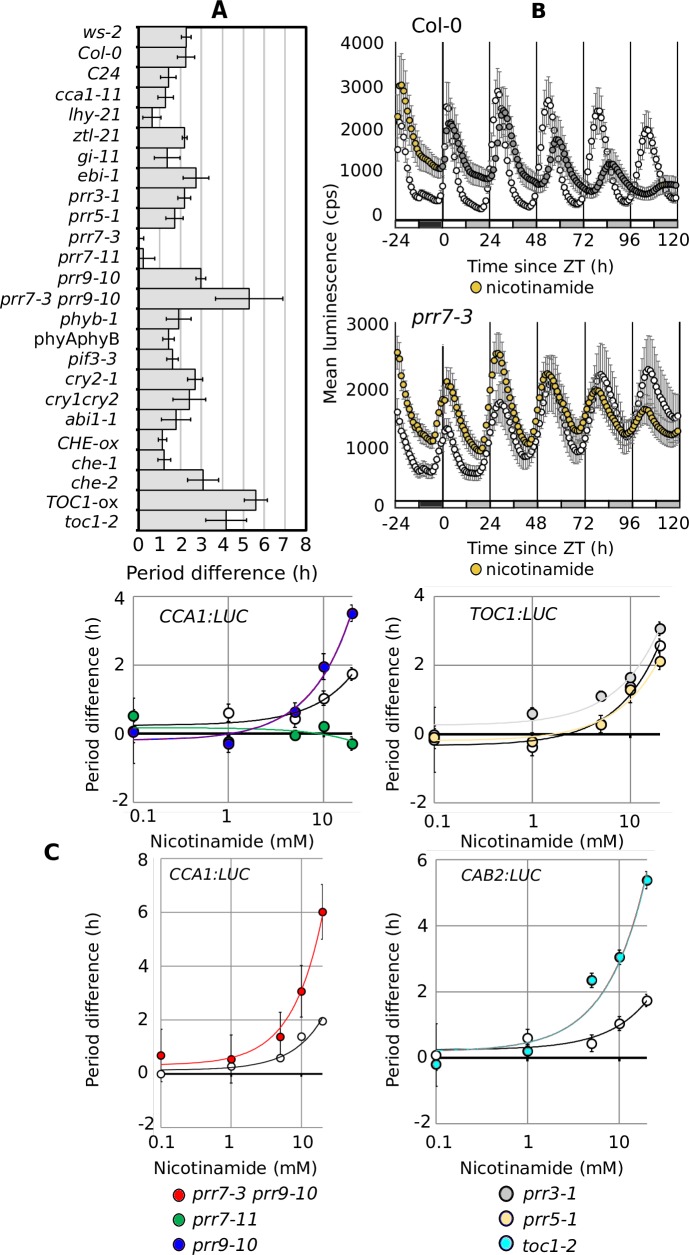
Reverse genetic analysis validates the prediction that *TOC1* and *PRR7* are associated with the effect of nicotinamide on circadian period. (**A**) The change in circadian period caused by 20 mM NAM (period difference) in circadian mutants measured using *CCA1*:*LUC*, *TOC1*:*LUC*, and *CAB2*:*LUC*. (**B**) *CCA1*:*LUC* activity for Col-0 and *prr7*-3 in the presence (yellow) and absence (white) of 20 mM NAM. (**C**) Dose response of circadian period to NAM for *prr7-11*, *prr9*-10, *prr7-3prr9-10*, *prr3*-*1*, *prr5-1* and *toc1*-*2*. Bars ±SD. N >16 from > 2 technical replicates. Open symbols indicate minus NAM. Mean time courses for these data are shown in S5 Fig. Statistical analysis is detailed in S6 Table.

To test the predictions that *TOC1*, *CRY2*, *CCA1* and *PRR7* are associated with the effect of NAM on the circadian oscillator, we experimentally investigated the sensitivity of circadian mutants to NAM. All mutants responded to NAM with increased circadian periods, with the exception of two independent lines of the same T-DNA insertion allele of *PRR7*, which were insensitive (*prr7-3* p > 0.95; *prr7-11* p > 0.95 Fig 5A and 5B; S5 Fig; S6 Table). The insensitivity of *prr7-11* to NAM was confirmed by measuring circadian rhythms of leaf movement (S6 Fig). *prr7*-*11* was not affected by NAM at any tested concentration, contrasting with a dose-dependent effect of NAM on circadian period in other *prr* mutants and associated backgrounds (R^2^ > 0.9; Fig 5C).

In contrast, *toc1*-*2* and *TOC1-*ox had significantly greater responses to NAM than wild type (Fig 5A; S6 Table). These results support our predictions that NAM induces dynamical changes specifically to *PRR7* and *TOC1*. No dramatic changes of period, however, were observed for *cry2-1* and *cca1-11*, suggesting that these might not contribute directly to the response to NAM.

Finally, derived from the nu-gap analysis, the possible change in the dynamical behavior of *PRR9* in mediating the effect of NAM on the clock was evaluated with a *prr7-3* and *prr9*-*10* double mutant. *prr7-3* and *prr9*-*10* had an epistatic interaction, with the double mutant responding to NAM by a 5.3 ± 1.6 h increase of period, more than either the insensitive *prr7-3* or the oversensitive *prr9-10* alone (Fig 5A). The epistasis of *prr9-10* to *prr7-3* was confirmed at all concentrations of NAM tested (Fig 5C).

### Nicotinamide-induced changes in period are associated with a blue light signaling pathway

The mutant analysis did not confirm the modeling dynamical perturbation of *CRY2* in the response to NAM. However, CRY2 is one of a pair of cryptochrome blue light photoreceptors and so mutant analysis might not be the most appropriate tool to investigate the role of the blue light photoreceptor. To investigate further we also investigated the role of blue light in the response to NAM using monochromatic light conditions. High frequency measurements of the circadian promoter fusions *PRR9*:*LUC*, *PRR7*:*LUC*, *TOC1*:*LUC*, *CCA1*:*LUC*, *LHY*:*LUC* and *GI*:*LUC* were collected in the presence or absence of 20 mM nicotinamide under constant blue or red light (S7 Fig).

**Fig 6:**
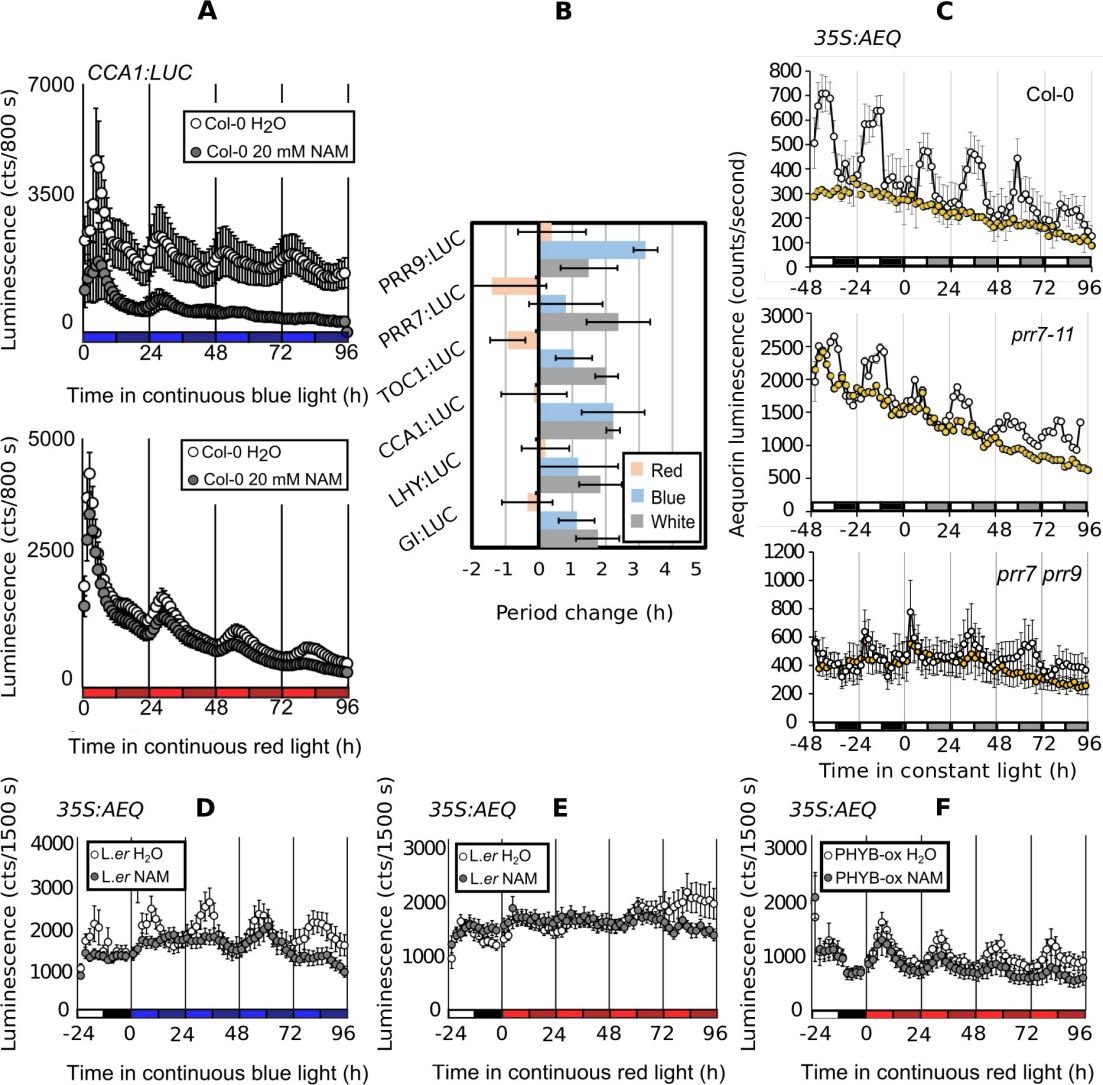
The effect of nicotinamide on circadian period requires blue light. (**A**) *CCA1*:*LUC* rhythms in monochromatic blue or red light ± 20 mM NAM. (**B**) The change in circadian period ± 20 mM NAM in constant white (grey) and monochromatic red or blue light for a range of reporters. (**C**) 20 mM NAM abolishes circadian rhythms of [Ca^2+^]_cyt_ in both Col-0, *prr7-11* and *prr7-prr9*. (**D**) Robust circadian rhythms of [Ca^2+^]_cyt_ in monochromatic blue light are abolished by 20 mM NAM. (**E**) Red light induced elevations of [Ca^2+^]_cyt_ early in the photoperiod are not abolished by nicotinamide. (**F**) *PHYB*-ox enhances circadian rhythms of [Ca^2+^]_cyt_ in monochromatic red light. Light conditions are indicated by the colored boxes on the X axes. White is red/blue mix, black is dark, monochromatic light is represented by the appropriate color with subjective night shaded darker than subjective day. NAM indicated by yellow. Bars are SD. n > 7.

In the absence of blue light, NAM was without effect on the circadian period or amplitude of *CCA1*:*LUC* (Fig 6A) and other promoter:luciferase fusions (Fig 6B). This demonstrates that input pathways associated with blue light are sensitive to NAM. Under blue light exposure, all promoter:luciferase fusions considered had an increase in period in the presence of NAM (Fig 6B). Under red light exposure, the period response was either negligible (*PRR9*:*LUC*, *CCA1*:*LUC*, *LHY*:*LUC*, *GI*:*LUC*) or negative (*PRR7*:*LUC*, *TOC1*:*LUC*). These results suggest that blue light increase the response of circadian period to NAM, while red light decrease its responsiveness.

Having previously proposed that the effects of NAM on the circadian system are due to the inhibition of the production of the Ca^2+^-agonist cADPR [12], we tested if the response to NAM of *prr7-11* is due to altered Ca^2+^ signaling. We investigated, therefore, the inhibitory effects of NAM on circadian [Ca^2+^]_cyt_ oscillations in *prr7-11* and in light signaling mutants in red and blue light. 20 mM NAM was equally effective in abolishing circadian rhythms of [Ca^2+^]_cyt_ in both Col-0, *prr7-11* and *prr7-3 prr9-10* (Fig 6C). This suggests either that there are multiple sites of action of NAM or that *PRR7* is downstream of the effects of NAM on [Ca^2+^]_cyt_.

In constant blue light, there were robust oscillations of [Ca^2+^]_cyt_ in plants with functional *CRY1* photoreceptors, being abolished in *cry1* and, *cry1cry2* but unaffected by *cry2*, *phototropins* and *Phy* loss-of-function mutants (Fig 6D, S8 Fig). Under blue light, NAM abolished [Ca^2+^]_cyt_ oscillations but did not reduce oscillations further in *cry1* or *cry1cry2* (Fig 6D, S9 Fig). High amplitude oscillations of [Ca^2+^]_cyt_ were dependent on blue light because in constant red light, [Ca^2+^]_cyt_ increased early in each cycle but without a subsequent decrease (Fig 6E; S8 Fig). This red light-induced increase in [Ca^2+^]_cyt_ was dependent on *PHYB* (S8 Fig).

To examine the role of *PHYB* further we measured [Ca^2+^]_cyt_ in *PhyB*-ox (Fig 6F, S8 Fig) and determined that in these plants [Ca^2+^]_cyt_ was rhythmic with a sinusoidal period of 25.0 ± 0.5 h in constant red light (Fig 6F). NAM was without effect on [Ca^2+^]_cyt_ in constant red light, even in the *PHYB*-ox background (Fig 6E and 6F, S6 and S7 Figs) demonstrating that blue light regulates circadian oscillations of [Ca^2+^]_cyt_ through a NAM-sensitive pathway. This pathway appears to be required for the major oscillatory dynamics of [Ca^2+^]_cyt_.

### Extension of DyDE to the rhythmic transcriptome

DyDE was further adapted to explore the rhythmic genome for additional targets for NAM and novel clock genes. For this purpose, models were computed between each pair of the 988 genes that were scored rhythmic in both untreated and NAM treated conditions, resulting in 2 million models corresponding to potential interactions.

We selected the models that exhibit the highest goodness of fit (over 80%) in both untreated and NAM-treated plants to minimize the identification of erroneous interactions and computed their nu-gap value to investigate dynamics affected by NAM. As a result, out of ten, two models only were retained with a nu-gap > 0.2. These models identified the regulation of *AT5G35970* (P-loop containing nucleoside triphosphate hydrolases superfamily protein) by *AT2G21860* (violaxanthin de-epoxidase-like protein) and the regulation of *ATG21660* (*GRP7/CCR2*) by *AT1G78600* (*LZF1/BBX22*) as being altered by NAM. The regulation of *AT5G35970* by *AT2G21860* may be important as *AT5G35970* is identified by DyDE as being a hub regulated by four circadian oscillator genes (S8 and S9 Tables). The second link is easier to explain because *GRP7* along with *GRP8* forms a slave oscillator driven by the circadian clock that regulates ABA responses [43]. *GRP7* is an RNA binding protein regulated by ADP ribosylation [44]. As the enzymes that perform ADP ribosylation are inhibited by NAD, this could suggest a role for nicotinamide inhibiting ADP ribosylation of an oscillator or slave oscillator component.

Then, the fitness threshold was released to 60% to further investigate novel clock components. For this purpose, we searched for those genes for which models can be computed from/to clock components (S8 Table). Models with a nu-gap value above 0.2 were discarded as a consistency criterion. Finally, candidates were ranked according to their connectivity with the clock. As a result, 20 high potential genes were isolated (S9 Table). The whole genome analysis of clock input and output hubs and the nu-gap analysis suggest interesting roles for previously characterized genes, including *AT3G47500* (*CYCLING DOF FACTOR3*) [45], *AT4G38960* (*BBX19*) [46], *AT1G78600* (*BBX22*) [47,48], *AT3G22840* (*CRY3*) [49], *AT1G28330* (*DRM1*), *AT2G33830* (*DRM2*) [50,51] and uncharacterized genes including *AT5G35970*.

## Discussion

The quantity and relevance of experimental measurements as well as *a priori* biological knowledge of the system are the two mains factors that determine the choice of appropriate model complexity. Here, we considered the problem of inferring the entry point of a treatment in an organism from limited time-series data (in this case, the circadian clock in Arabidopsis). For this purpose, we used simple dynamical models to capture gene regulatory dynamics and compare those under different scenarios without making *a priori* assumptions on the structure of the network. Subsequently, we showed that simple dynamical models have the potential to identify crucial dynamical perturbations for complex systems such as the circadian clock. However, it should be stressed that, as for the sole purpose of identifying the topology of the underlying network, our method competes well with the current state-of-the-art of network inference strategies. To provide a more comprehensive picture, we further conducted a comparative analysis of the network reconstruction performances of our algorithm with four different methodologies [25,28,30,52] on a widely used circadian benchmark dataset generated from the Pokhilko 2010 model [25,53]. The simulations were carried to replicate the experimental conditions of our dataset (constant light, 48h of observational time, 4h sampling rate) [54,55]. S10 Fig and S10 Table displays the respective performance of each algorithm in terms of the resulting Area Under the ROC Curve and the Precision-Recall Curve. Precision-Recall curves allow for a more accurate picture of algorithms performances for sparse GRNs (such as the plant circadian clock) and are commonly used for such task. As a result, it can be seen that the performances of our methodology approach those of an adaptation of the best performer of the Dialogue for Reverse Engineering Assessments and Methods (DREAM) [26] to time-series data: the semi-parametric method dynGENIE3. The nonlinear parametric approach (GESBL) and the semi-mechanistic methodology (iCheMA) both seem to underperform. However, the non-parametric approach (GDPM) significantly outperformed all methods provided. This result is consistent with the currently available literature, which seems to favour the use of nonparametric nonlinear equations for the inference of network topology from short-time series data. However, as mentioned above, comparison of nonlinear dynamics is not trivial, and require further mathematical developments. Hence, as a future work, it would be interesting to address the development of such suitable mathematical tools.

We further devised a learning algorithm to capture the specific pattern of oscillating wave forms of genes affected by NAM. Since the period of oscillations of central clock genes increases from 24 (wildtype) to roughly 28 (NAM) hours, we focused on those genes. For a relatively small number of genes, DyDE efficiently narrowed down possible targets of NAM that could then be verified experimentally. Since it is likely that other genes may be targets of NAM, we further applied DyDE to all 988 circadian genes that were scored rhythmic in both untreated and NAM treated conditions.

DyDE identified important changes in the regulatory dynamics of *PRR7*, *TOC1*, *CCA1* and the blue light photoreceptor, *CRY2*, resulting from the treatment of plants to NAM as well as suggesting a mediating role of *PRR9*. Mutants analysis confirmed DyDE predictions of altered activity of *PRR7*, *TOC1* and *PRR9* and blue/red light experiments demonstrated that the effect of NAM is blue light dependent. The latter also demonstrated that blue light regulates circadian oscillations of [Ca^2+^]_cyt_ through a NAM-sensitive pathway.

The involvement of *PRR7* with the dynamic adjustment of circadian period in response to nicotinamide, revealed by the insensitivity of *prr7-11* and *prr7-3* to NAM and confirmed by leaf movements analysis, is interesting because *PRR7* is also required for the response of the circadian oscillator to sugars [10,56]. *PRR7*, however, is not a direct target for NAM in the circadian oscillator because *PRR7* is not required for the response to NAM, as demonstrated by the hyper-sensitivity to NAM of the *prr7-3 prr9-10* double mutant. Together, the insensitivity of *prr7-3* and *prr7-11* to NAM and hypersensitivity in the *prr7-3 prr9-10* double mutant indicates that *PRR7* and *PRR9* regulate a component or pathway influenced by NAM and that *PRR7* might act upstream of *PRR9* in this regulation. The levels of expression of *PRR7* and *9* appear to regulate the pace of the circadian oscillator through feedback with CCA1/LHY and by acting as toggle switching the oscillator from a morning state when CCA1/LHY are high to an evening state when TOC1 is high [57,58].

**Fig 7:**
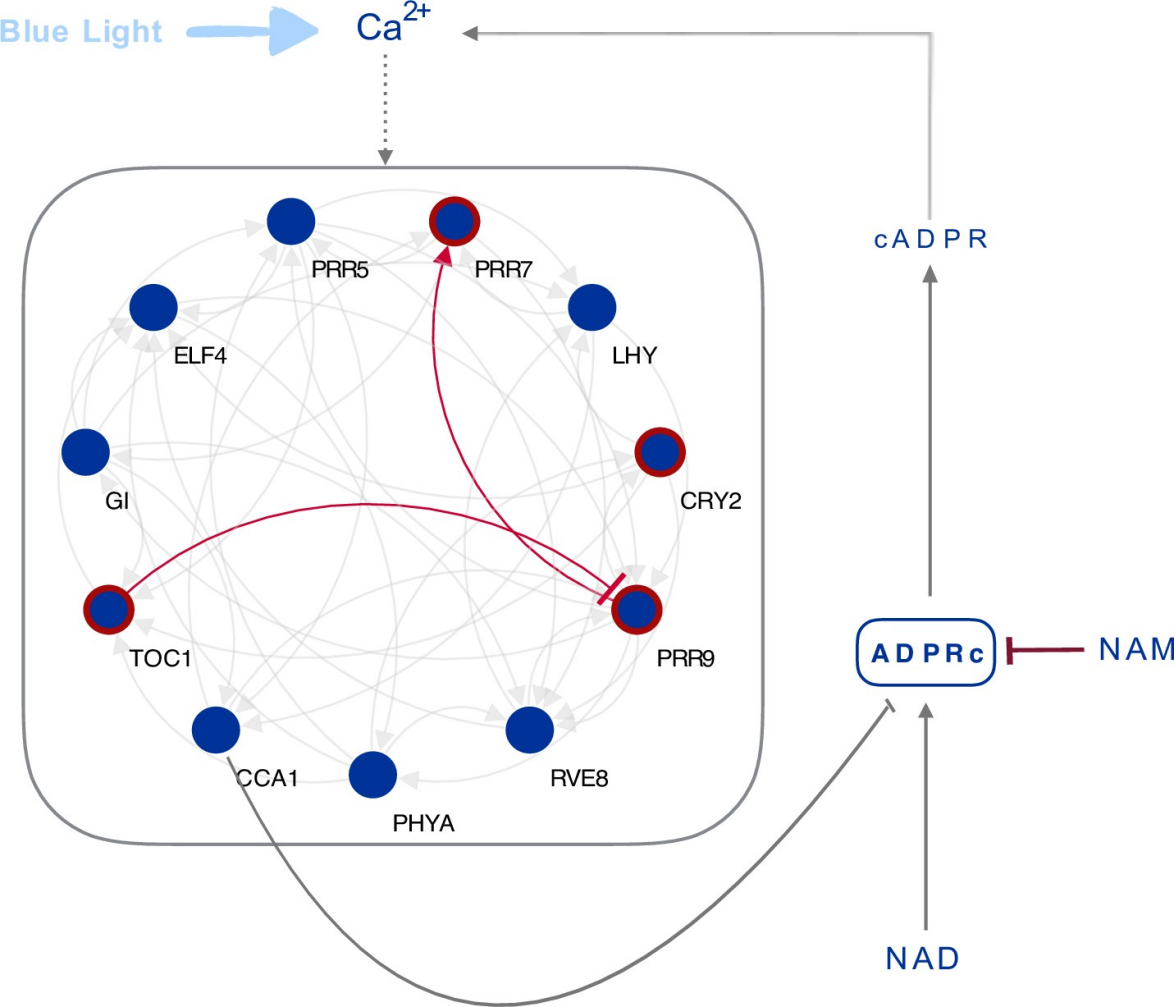
A blue light dependent module regulates the response of the circadian oscillator to NAM. NAM might regulate the circadian oscillator through regulation of cADPR dependent circadian oscillations of [Ca^2+^]. *CCA1* is a repressor of ADPRc. ADPRc generation of cADPR and [Ca^2+^] oscillations is inhibited by NAM. Both the effects of NAM on the circadian oscillator and circadian oscillations of [Ca^2+^]_i_ are blue-light dependent. The regulation of [Ca^2+^] on the circadian oscillator is indicated by a dotted line. NAM could also regulate the circadian oscillator by Ca^2+^-independent events. We determined that the NAM-induced changes in circadian period are mediated principally by the interaction between *PRR7* and *PRR9*, as well as *TOC1*.These interactions are shown in red in the model.

Additionally, the blue-light dependency of both circadian oscillations of [Ca^2+^]_cyt_ and NAM regulation of circadian period might suggest that Ca^2+^ is associated with the response of the oscillator to NAM. Furthermore, we recently reported that CALMODULIN-LIKE 24 (CML24), is a Ca^2+^-dependent regulator of circadian period and that its effects are NAM sensitive [59]. A caveat to this argument is that our methodology identified *CRY2* regulation of the transcriptional network being altered by NAM but the circadian oscillations of [Ca^2+^]_cyt_ were dependent on *CRY1*. NAM can also affect the oscillator through Ca^2+^-independent mechanisms [14]. We propose that a module of circadian oscillator components *PRR7* and *9*, *TOC1* and a Ca^2+^ signaling network contribute to the blue light-dependent response of the circadian oscillator to NAM that regulates circadian period (Fig 7).

Then, extension of DyDE to the whole circadian genome has also identified components outside the core oscillator that might also be involved in response to NAM, including the regulation of *GRP7/CCR2* by *LZF1/BBX22* and these will be candidates for future investigation. Remarkably, five genes out of 22 that were isolated in our genome analysis are known to interact with circadian regulators (*BX19*, *CYCLING DOF FACTOR3*) [45,46], have been previously implicated in circadian regulation (*BBX22*) [47,48], in blue light signaling (*CRY3*) [49] or are being downregulated by ABA/cADPR (*GRP7*, *BBX19*, *BBX22*) [43,44,60]. This result is encouraging and opens the door to the identification of novel drivers of circadian rhythms in Arabidopsis.

Overall, we suggest that the description of gene regulatory dependencies and the quantification of changes in dynamics computed by DyDE provide reliable hypotheses for the investigation of drug targets in complex gene regulatory networks, which has a broad range of applications in systems biology.

### Experimental procedures

#### Plant material and growth conditions

*Arabidopsis thaliana* C24 ecotype untreated plant carrying p*CHLOROPHYLL A/B BINDING-PROTEIN2*:*LUCIFERASE* (*CAB2*:*LUC*) and p*35SCAMV*:*AEQUORIN* (*35S*:*AEQ*) and photoreceptors mutants carrying *35S*:*AEQ* were previously reported [61]. All other plant lines were previously reported [10]. *Prr7-3* and *prr7-11* are the same T-DNA insertion loss-of-function mutation being independently characterised as *prr7-3* [62] and *prr7-11* [63]. These were maintained as separate populations. Growth conditions were as previously described [10].

#### Microarray datasets

C24 seeds were grown for 10 days. On the 11^th^ day the seedlings were exposed to constant light (70 μmol m^-2^ s^-1^). 50% of C24 plants were treated with 50 mM NAM (Sigma, U.K.) every two hours as previously described [41]. Aerial tissue from 100 seedlings was sampled every 4 hours from ZT 49 to 93. RNA extraction was performed as described [12]. Three independent experiments were carried out and 100 ug of RNA from two of these was sent to NASC (Nottingham, UK) for microarray analysis using the ATH1 array. Note that *PRR9* and *LUX* probes on the ATH1 microarray also measure the expression levels of *AT2G46670* and *AT5G59570*, respectively. Comparing other datasets [40] shows that the probe with *PRR9* agrees with *PRR9* on other datasets while the one with *LUX* does not. Hence, we used this probe to represent *PRR9*, but not *LUX*.

All modeling and analysis was carried out in MATLAB unless stated otherwise. Microarray data was normalised using gcRMA. Go enrichment was determined using Gorilla [64]. Promoter motif enrichment was calculated using AtCOECIS [65].

#### Validation of microarray data sets

The microarrays were validated by RT–qPCR using aliquots of the RNA extracted for microarray hybridization as described previously [10]. *CCA1* and *TOC1* were analysed and quantified as detailed previously [10].

#### Luminescence time course experiments

Photon counting imaging of aequorin or luciferase activity in constant light of 60–70 μmol^-2^ s^-1^ monochromatic blue, monochromatic red or equal blue-red LED light was performed as described [12]. Circadian period estimates were calculated using the MS Excel BRASS plugin (http://amillar.org/downloads).

## Supporting information

S1 Fig Detrended time-series of circadian transcript in both untreated and NAM condition. Data were gathered for 44 hours every 4 hours, 2 replicates, starting from 49 hours after the switch to constant light (i.e., third day of constant light). Data showed are detrended, so that the rhythmic pattern is clear. *LUX* does not appear on this list, as the probe also measured the expression of *AT5G59570*. *CRY1*, *PHYB*, *ELF3*, *ZTL* and *CHE* were not considered for the network inference step.
(EPS)

S2 Fig Assessment of circadian regulated transcripts from both the learning methodology and standard tools. **(A)** Results correspond to untreated plants. The trained algorithm, COSOPT [33] and JTK [32] respectively identified 3859, 1856 and 3698 circadian regulated transcripts. JTK and the trained algorithm identified most of the genes labelled as periodic by COSOPT (resp. 87% and 81% of them). The rhythmicity of 75% of the genes labelled rhythmic by JTK was confirmed by the learning strategy. **(B)** Results corresponding to NAM-treated plants. The rhythmicity of 60% of the genes labelled rhythmic by JTK was confirmed by the learning strategy while 1636 novel genes were identified as rhythmic with a typical non-sinusoidal profile.
(EPS)

S3 Fig Coverage and false positive curves of the known regulatory links involved in the circadian oscillator of Arabidopsis Thaliana, as inferred by DyDE. In DyDE, linear Ordinary Differential equations (ODEs) of order one are computed between each pair of genes to describe the dynamics of the whole system. To be further considered as a good approximation of the dynamics involved, each dynamical model needs to pass a validation criterion based on its agreement to the data (i.e. a user-defined threshold on the goodness of fit). Decreasing the fitness threshold leads to a better coverage (upper panel) of the system dynamics but increase the amount of false positives (lower panel). The coverage describes the amount of links inferred over the amount of total true links in the system (as defined by Fogelmark et al, 2014). The number of false positive corresponds to links that are not represented in Fogelmark et al. The maximum amount of possible false positives is 32, while the total amount of links in the true system is of 40. The threshold of 46% (represented by a red cross) is chosen for this analysis with a coverage of 70% (which corresponds to 28 true positives and 21 false positives).
(EPS)

S4 Fig Networks inferred by DyDE in both untreated and NAM-treated plants, with the regulation loss networks. **(A)** Network inferred in absence of NAM. **(B)** Network inferred in presence of NAM. **(C)** Regulation loss network. **(D)** Common regulation network. On these graphs, genes are organized by their peaking time during diel cycles. Genes that are represented by a yellow filled circle peak in the morning, dark brown filled circles stand for an evening peaking time and grey for a night peaking time. White filled circles correspond to photoreceptors. Networks are made of the conjunction of identified LTI models for which respective fitness values are above 46%. Blue arrows correspond to activation whereas red arrows correspond to an identified inhibition (S3 Table). On **(D)**, the highlighted links represent the models with the five highest nu-gap values.
(EPS)

S5 Fig The effect of nicotinamide on circadian rhythms in circadian, light and ABA signaling mutants. Rhythms of luciferase activity were measured in the presence (grey) or absence (white) of 20 mM NAM. Col-0 *CCA1*:*LUC*, *Col-0 TOC1*:*LUC*, WS *CAB*:*LUC*^*+*^ and C24 *CAB*:*LUC* backgrounds are shown with respective mutants. Traces represent mean values of n > 16 biological replicates from > 2 technical replicates in the presence (grey) or absence (clear) of 20 mM NAM
(EPS)

S6 Fig Circadian rhythms of leaf movement are insensitive to nicotinamide in *prr7-11*. Rhythms of leaf movement were measured in constant white light for 120 h in the presence (yellow) or absence (white) of 20 mM NAM. *n* = 22 Col-0, and *n =* 30 *prr7-11*.
(EPS)

S7 Fig Circadian rhythms of *CCA1:LUC, LHY:LUC, PRR7:LUC, PRR9:LUC, TOC1:LUC and GI:LUC* activity in constant red and blue mixed light, constant red or constant blue light (70 umol m-2 s-1). Grey indicates the presence and white indicates the absence of 20 mM NAM. Light conditions indicated by the colored boxes on the X axes. White is red/blue mix, monochromatic light is indicated by the appropriate color with subjective night shaded darker than subjective day.
(EPS)

S8 Fig Phytochrome and cryptochrome modulate [Ca^2+^]_cyt_ under monochromatic light. 35S:AEQ luminescence measured over 96 hours in constant 65 umol m^-2^ s^-1^ monochromatic red or blue light in light signaling mutants. Bars on the X axis indicate red and blue mixed light (white), dark (black), monochromatic red (red), or monochromatic blue (blue). Darker boxes indicate subjective night in constant light. N = 8. Error bars SEM.
(EPS)

S9 Fig Blue light-sensitive circadian [Ca^2+^]_cyt_ oscillations are inhibited by nicotinamide. 35S:AEQ luminescence measured over 96 hours in constant 65 umol m^-2^ s^-1^ monochromatic red or blue light in light signaling mutants in the presence (grey circles) and absence (open circles) of 20 mM NAM. Bars on the X axis indicate light treatment of red and blue mixed light (white), dark (black), monochromatic red (red) or monochromatic blue (blue). Darker boxes indicate subjective night in constant light. n = 8. Error bars SEM.
(EPS)

S10 Fig Comparative analysis of the network reconstruction accuracy of our algorithm with state-of-the-art network inference methodologies. The Area Under the ROC Curve and the Precision-Recall Curve are shown for each algorithm and simulation run. For this comparison, only 10 stochastic simulations were carried to limit the overall computation time of methodologies that involve a Markov Chain Monte Carlo (MCMC) sampling scheme. Each box on these graphs represents the 50^th^– 95^th^ percentile around the median. Dynamical GENIE3 (dynGENIE3, [28]) is an adaptation of the GENIE3 [S1] method for time series data, which was the best performer of the DREAM4 Multifactorial Network challenge and the DREAM5 Network Inference Challenge [26,S2]. Like our approach, the temporal evolution of genes in dynGENIE3 is based on an ODE model. However, in this specific case, the transcription function in each ODE is represented by ensembles of regression trees and is therefore not fixed a priori. This semi-parametric approach provides a greater flexibility to the inference framework but complicates the comparison of dynamical properties between experimental conditions. In contrast, Continuous-time Gaussian process dynamical model (GPDM) is a non-parametric, Gaussian process-based GRN inference algorithm for which the performances were compared to the best performers of the DREAM challenge and consistently shown superior in dealing with short time series data generated from the 10-genes challenge [30]. A parametric approach, so-called Sparse Bayesian Learning (GSBL) has also been investigated to identify a sparse representation of GRN topology with nonlinear multivariate ARX models and showed promising performances on a repressilator model of gene regulation [52]. Finally, a semi-mechanistic model based on gradient matching and nonlinear Hill-type transcription function such as the iCheMA [25] algorithm have been developed and was revealed as the best performer of a set of established state-of-the-art network reconstruction methods applied to the inference of circadian-type regulatory networks. Our algorithm reached an AUROC of 0.6426 +- 0.0448 and an AUPREC of 0.5124 +- 0.049. dynGENIE3 obtained an AUROC of 0.6709 +- 0.0427 and an AUPREC of 0.512 +- 0.049. GPDM obtained an AUROC of 0.7119 +- 0.0538 and an AUPREC of 0.6759 +- 0.0745. GESBL obtained an AUROC of 0.5801 +- 0.0542 and an AUPREC of 0.4999 +- 0.707 while iCheMA obtained an AUROC of 0.5523 +- 0.0874 and an AUPREC of 0.2848 +- 0.0397. Details of the results for each run are presented on the S10 Table.
(EPS)

S1 Table Circadian transcriptomes of untreated and nicotinamide-treated plants. Circadian time-series for the transcripts on the ATH1 microarray for the untreated and nicotinamide-treated (NAM) datasets are presented on three separate sheets. Gene lists are provided describing the genes that were identified by PEAL to be circadian-regulated, along with the estimated period, amplitude and phase, and confidence of estimation for C24, NAM and *toc1-1*. The final two sheets describe the genes common between the C24 and NAM, and the C24 and *toc1-1* circadian-regulated subsets.
(XLSX)

S2 Table GO enrichment terms of transcripts rhythmic. The tables list the most enriched terms in the rhythmic transcripts, split in to the three ontologies: Biological process, Molecular function, and Cellular component. The analysis is split in eight categories, each corresponding to a column in the table: GO term (unique identifier in the GO database), GO description, p-value (likelihood of enrichment), False Discovery Rate q-value, a measure of Enrichment, a link to the full list of enriched genes, and whether the GO term is also enriched in the NAM datasets.
(XLSX)

S3 Table Details of the dynamical interactions inferred by DyDE between the genes of the *Arabidopsis thaliana* circadian oscillator. The tables describe the connections modelled for the circadian oscillator in the untreated plants, NAM treated, regulation loss and common networks. Network connections are described gene by gene and a comparison with the known regulatory interactions is provided. The fitness value is displayed, as well as the sign of the regulation (activation or inhibition).
(XLSX)

S4 Table Sorted v-gap values corresponding to common links between untreated and NAM. Nu-gaps values computed for each link inferred in both untreated and NAM-treated networks. This table ranks the nu-gap values from the largest to the smallest.
(XLSX)

S5 Table Connectivity loss corresponding to each gene, for untreated and NAM-treated networks. Values are displayed for a fitness threshold of 46%.
(XLSX)

S6 Table The effect of nicotinamide on circadian period in circadian oscillator mutants. The period of circadian rhythms of luciferase activity were measured in the presence absence of 20 mM NAM in Col-0 *CCA1*:*LUC*, *Col-0 TOC1*:*LUC*, WS *CAB*:*LUC*^*+*^ and C24. *CAB*:*LUC* backgrounds are reported with the respective mutants. n > 16 biological replicates from > 2 technical replicates.
(XLSX)

S7 Table Cost functions and parameters optimization of the P2012 model. These tables sort the cost function scores obtained by sequentially optimizing a single and two parameters simultaneously to the best fit of TOC1 and CCA1 time-series profile in the presence of NAM. Best performances for single parameter tuning are achieved for m14 and m15 respectively while the best performances for two simultaneous parameters are obtained through a mixture of LHY and PRR7 parameters.
(XLSX)

S8 Table Extension of the DyDE methodology to the whole genome: List of potential novel candidates involved in clock regulation. The list of models that have been validated (> 60% fitness, nu-gap < 0.2) between the genome and the known clock genes. The lists are split in four sheets, displaying separately the analysis of the genome to the main clock genes and vice versa. The genes are sorted in alphabetical order. Their function and the sign of the identified regulation are provided, as well as the count of appearance of each gene in their respective list.
(XLSX)

S9 Table Summary of the novel potential clock genes identified. The list of genes identified as input/output hubs to the clock as well as their functions is represented together with their potential regulation by cADPR.
(XLSX)

S10 Table Details of the performances of state-of-the-art network inference methodologies against our approach. This table summarize the Area Under the ROC Curve and the Precision-Recall curve obtained by each algorithm, for each run. Highlighted is the best performer for each run.
(XLSX)
